# A Rapid Method for Generating Infectious SARS-CoV-2 and Variants Using Mutagenesis and Circular Polymerase Extension Cloning

**DOI:** 10.1128/spectrum.03385-22

**Published:** 2023-03-06

**Authors:** Beom Kyu Kim, Won-Suk Choi, Ju Hwan Jeong, Sol Oh, Ji-Hyun Park, Yu Soo Yun, Seong Cheol Min, Da Hyeon Kang, Eung-Gook Kim, Hojin Ryu, Hye Kwon Kim, Yun Hee Baek, Young Ki Choi, Min-Suk Song

**Affiliations:** a Department of Microbiology, Chungbuk National University, College of Medicine and Medical Research Institute, Cheongju, Chungbuk, Republic of Korea; b Department of Biochemistry, Chungbuk National University, College of Medicine and Medical Research Institute, Cheongju, Chungbuk, Republic of Korea; c Department of Biological Sciences and Biotechnology, College of Natural Science, Chungbuk National University, Cheongju, Republic of Korea; d Center for Study of Emerging and Re-emerging Viruses, Korea Virus Research Institute, Institute for Basic Science (IBS), Daejeon, Republic of Korea; Emory University School of Medicine

**Keywords:** SARS-CoV-2, infectious clone, mutagenesis, CPEC, reverse genetics

## Abstract

The appearance of SARS-CoV-2 variants in late 2020 raised alarming global public health concerns. Despite continued scientific progress, the genetic profiles of these variants bring changes in viral properties that threaten vaccine efficacy. Thus, it is critically important to investigate the biologic profiles and significance of these evolving variants. In this study, we demonstrate the application of circular polymerase extension cloning (CPEC) to the generation of full-length clones of SARS-CoV-2. We report that, combined with a specific primer design scheme, this yields a simpler, uncomplicated, and versatile approach for engineering SARS-CoV-2 variants with high viral recovery efficiency. This new strategy for genomic engineering of SARS-CoV-2 variants was implemented and evaluated for its efficiency in generating point mutations (K417N, L452R, E484K, N501Y, D614G, P681H, P681R, Δ69-70, Δ157-158, E484K+N501Y, and Ins-38F) and multiple mutations (N501Y/D614G and E484K/N501Y/D614G), as well as a large truncation (ΔORF7A) and insertion (GFP). The application of CPEC to mutagenesis also allows the inclusion of a confirmatory step prior to assembly and transfection. This method could be of value in the molecular characterization of emerging SARS-CoV-2 variants as well as the development and testing of vaccines, therapeutic antibodies, and antivirals.

**IMPORTANCE** Since the first emergence of the SARS-CoV-2 variant in late 2020, novel variants have been continuously introduced to the human population, causing severe public health threats. In general, because these variants acquire new genetic mutation/s, it is critical to analyze the biological function of viruses that such mutations can confer. Therefore, we devised a method that can construct SARS-CoV-2 infectious clones and their variants rapidly and efficiently. The method was developed based on a PCR-based circular polymerase extension cloning (CPEC) combined with a specific primer design scheme. The efficiency of the newly designed method was evaluated by generating SARS-CoV-2 variants with single point mutations, multiple point mutations, and a large truncation and insertion. This method could be of value for the molecular characterization of emerging SARS-CoV-2 variants and the development and testing of vaccines and antiviral agents.

## INTRODUCTION

Severe acute respiratory syndrome coronavirus 2 (SARS-CoV-2) is one of three recognized, significant respiratory disease-causing viruses of the *Coronaviridae* family, together with severe acute respiratory syndrome coronavirus (SARS-CoV) and the Middle East Respiratory Syndrome Coronavirus (MERS-CoV). Since its first emergence in December 2019, coronavirus disease (COVID-19) has been responsible for more than 554 million cumulative human cases and more than 6.3 million recorded deaths worldwide (1). To minimize the enormous health hazards and global economic impacts of COVID-19, researchers have undertaken systematic genomic epidemiological investigations of SARS-CoV-2 (2–4).

Although all viruses can readily mutate, RNA viruses usually mutate faster than DNA viruses, with single-stranded viruses mutating more rapidly than double-stranded viruses (5). Among single-stranded RNA viruses, coronaviruses have moderate-to-high mutation rates (6). Combined with the current widespread distribution and circulation of SARS-CoV-2, the emergence of SARS-CoV-2 variants, which pose an increased risk to global public health, has prompted the tracking and characterization of specific variants of concerns (VOCs) and variants of interests (VOIs) (7). In December 2020, a novel variant with enhanced transmissibility, termed SARS-CoV-2 Alpha B.1.1.7 lineage (VOC-202012/01), was identified in the United Kingdom (4, 7–10). This variant was characterized as having substantial genetic changes specifically in the spike (S) region that might impact the pathogenicity of the virus and consequent severity of the disease (7–10). Two more VOCs—the Beta B.1.351 lineage in South Africa and Delta (B.1.617.2) from India (2, 7–9, 11)—were also first reported in 2020. By early 2021, another VOC, the Gamma variant of the P.1 lineage, was reported in Brazil. And in November 2021, the Omicron B1.1.529 lineage was reported in multiple countries (7). As of July 2021, the World Health Organization has described four other variants of interest: Eta of B.1.525, Iota of B.1.526, Kappa of B.1.617.1, and Lambda of C.37 lineage, each predicted to carry an increased risk to global public health owing to their associated impacts on virulence, host transmissibility, and antibody susceptibility (7, 12).

Because these VOCs and VOIs continue to pose threats to existing vaccines and antiviral and antibody drugs, it is important to further investigate the innate viral properties of these variants to establish a scientific basis for future public health policies and disease-prevention strategies (2, 9). As an alternative to current variant experimental studies, which remain restricted to inferring variant isolates from patient samples or the use of pseudo-viruses (12), researchers have developed various genomic approaches employing reverse genetics systems to engineer SARS-CoV-2 (4, 13). These include an *in vitro* ligation approach that uses bacterial artificial chromosomes to assemble cDNA fragments into a full clone of SARS-CoV-2 (14–18), and a yeast-based synthetic genomic platform using transformation-associated recombination (TAR) cloning, which was used to recombine seven or nine overlapping DNA fragments (19, 20). With the rampant emergence of variants comes a continuing need to rapidly characterize variant mutation profiles (12). However, the generation of various mutant viruses for use in characterizing genetic mutations that occurred in the viral genome remains a tedious and time-consuming process. The recently introduced circular polymerase extension reaction (CPER) method, which is capable of amplifying SARS-CoV-2 cDNA, promises rapid generation of viral mutants (21, 22). However, the method lacks the capacity to verify amplified products for errors prior to transfection, potentially introducing PCR-derived mutations in the recombinant virus.

Here, we utilize the circular polymerase extension cloning (CPEC) method (23, 24), a simplified sequence-independent cloning approach that relies on the polymerase extension mechanism, to regenerate SARS-CoV-2 viruses via reverse genetics. This method simplifies the cloning steps and is capable generating a homogeneous viral population but, unlike CPER, still requires steps to generate infectious clones in the plasmid vector through bacterial culture and sequence verification before transfection. With improvements in cloning accuracy and efficiency, CPEC was applied to the assembly of a three-fragment subclone construct, resulting in the successful recovery of a whole-genome SARS-CoV-2 infectious clone and diverse variants. Combining our CPEC and mutagenesis scheme using three-fragment subclone constructs yields a simpler, uncomplicated, and versatile “one-pot” cloning approach for generating full-length infectious clones and recovering wild-type and engineered SARS-CoV-2 variants. This improved strategy for genomic engineering of SARS-CoV-2 variants was implemented and evaluated for its efficiency in generating point mutations, multiple mutations, and a large truncation and insertion. Rescuing SARS-CoV-2 variants for further application of *in vitro* and *in vivo* studies will aid in the genetic characterization of these variants and enhance our understanding of virus evolution.

## RESULTS

### CPEC approach for generating recombinant SARS-CoV-2 virus.

**FIG 1:**
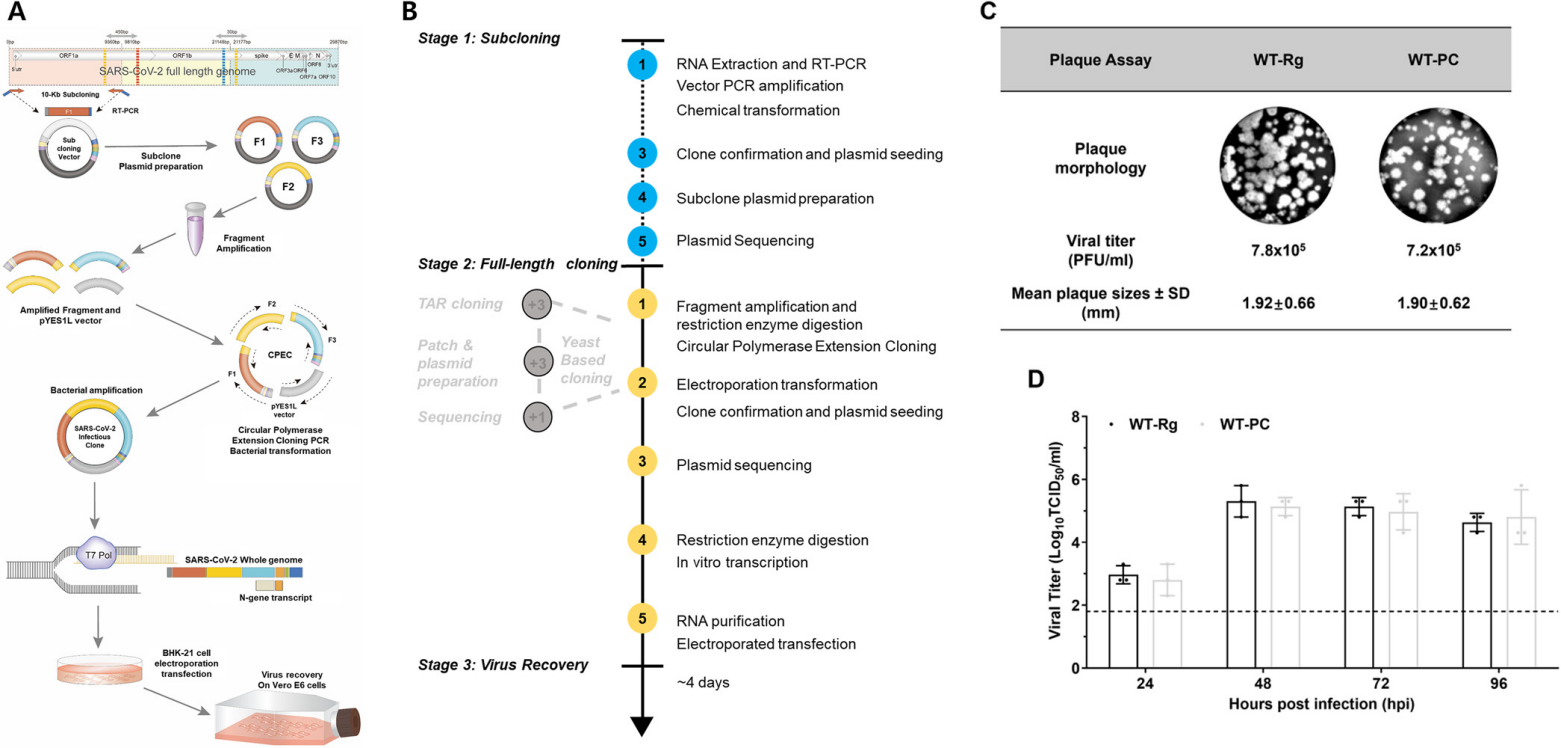
Overall scheme for the three-fragment subclone/CPEC approach for generating SARS-CoV-2 infectious clones and recovering SARS-CoV-2 virus. (A) Schematic diagram depicting the amplification of the whole genome of SARS-CoV-2, divided into three fragments (8.7 to 11.8 kb each), using CPEC; ligation and *in vitro* transcription of the assembled plasmid of the SARS-CoV-2 whole genome; recovery of intact virus by direct electroporation of *in vitro* transcripts into BHK-21 cells; and subsequent overlay onto Vero-E6 cells. (B) Time efficiency of the process. (C) Plaque formation. (D) *In vitro* growth assays, performed to assess phenotypic characteristics and growth of recovered SARS-CoV-2 viruses.

To confirm the utility of the CPEC method for reconstructing SARS-CoV-2 infectious clone, we designed primers to synthesize three cDNA fragments of 8.7 to 11.8 kb in size (Fig. 1A, B). Viral RNA extracted from the first passage of virus grown in Vero E6 cells was used for generating cDNA fragments by RT-PCR (Table 1). Each fragment was in-fused and subcloned individually into a designed pUC19 plasmid vector (Fig. S1A). The SARS-CoV-2 subcloning vector was modified to maintain the genomic fragment of SARS-CoV-2 and facilitate mutagenesis of the viral genome (Fig. S1B). The subcloning vector is equipped with two multirestriction endonuclease regions containing EagI, *Asi*SI, and ApaI sites; a T7 promoter for *in vitro* RNA transcription; two self-cleaving ribozymes, hammerhead (Hh) and hepatitis delta virus (HdV) ribozymes; and a T7 terminator (Fig. S1). Before assembly, each subclone was amplified using primers designed to contain a 15-bp extension of the 5′- or 3′-end sequence of the linearized subcloning vector (Table 1) for PCR generation of the three target fragments (8.7 to 11.8 kb each). After purification, the three genomic fragments of SARS-CoV-2 were assembled with a pYES1L vector using the CPEC cloning method. The generated PCR products can be directly processed for transformation into competent bacterial cells by electroporation without ligation or purification.

**TABLE 1:** Primers for SARS-CoV-2 subcloning and circular polymerase extension cloning (CPEC)

Primer name:	Primer code	Sequence (5′ to 3′)	Base pair	Comment:
For amplification of SARS-CoV-2 cDNA				
cDNA synthesis F1 primer	RT-1	GTACCAGCTGCTTGTGCTGTTTGCCTGTCAACAA	34	Primer for synthesis of SARS-CoV-2 F1 cDNA fragments
cDNA synthesis F2 primer	RT-2	CTACATGGCCATCTTTACACCAAAGCATAAATGAAATTTCTGTAT	45	Primer for synthesis of SARS-CoV-2 F2 cDNA fragments
cDNA synthesis F3 primer	RT-3	GTCATTCTCCTAAGAAGCTATTAAAATCAC	30	Primer for synthesis of SARS-CoV-2 F3 cDNA fragments
For amplification of SARS- CoV-2 subcloning fragments and vector				
Subcloning F1 insert F	SP-1	GAGTCTAGACTCCGTCATTAAAGGTTTATACCTTCCCAGGTAACAA	46	Primer sets for amplifying SARS-CoV-2 Fragment 1 insert
Subcloning F1 insert R	SP-2	GTCATTCTCCTAAGAATCACATGTCTTGGACAGTAAACTACGTCATCAAGCCAA	54	
Subcloning F2 insert F	SP-3	GAGTCTAGACTCCGTCACCACTAATTCAACCTATTGGTGCT	41	Primer sets for amplifying SARS-CoV-2 Fragment 2 insert
Subcloning F2 insert R	SP-4	GTCATTCTCCTAAGAACAACACCATCAACTTTCTTATAATAATTGAACTGTGTT	54	
Subcloning F3 insert F	SP-5	GAGTCTAGACTCCGTCCAATTATTATAAGAAAGTTGATGGTGTTGTCCAACAATT	55	Primer sets for amplifying SARS-CoV-2 Fragment 3 insert
Subcloning F3 insert R	SP-6	GTCATTCTCCTAAGAAGCTATTAAAATCACATGGGGATAGCACTACT	47	
Subcloning vector F	SP-7	TCTTAGGAGAATGACAAAAAAAAAAAAAAAAAAAAAAAAAAAAAAAAAAAGGCCGGCATGGTCCCA	81	Primer sets for amplifying the subcloning vector
Subcloning vector R	SP-8	GACGGAGTCTAGACTCCGTTTCGTCCTCACGGACTCATCAG	41	
For amplification of insert and vector for CPEC				
CPEC F1 insert F	CP-1	CGCTGATACCGCCGCGGGCCCTAATGCGATCGCATACGG	39	Primer sets for amplifying Fragment 1 for CPEC
CPEC F1 insert R	CP-2	GTGCACAGCGCAGCTTCTTCAAAAGTACTAAAGGAAACA	39	
CPEC F2 insert F	CP-3	ACCACTAATTCAACCTATTGGTGCTTTGGACATATCAGC	39	Primer sets for amplifying Fragment 2 for CPEC
CPEC F2 insert R	CP-4	ACAACACCATCAACTTTCTTATAATAATTGAACTGTGTT	39	
CPEC F3 insert F	CP-5	CAATTATTATAAGAAAGTTGATGGTGTTGTCCAACAATT	39	Primer sets for amplifying Fragment 3 for CPEC
CPEC F3 insert R	CP-6	TTAACTGCGGCGAGGGCGATCGCTATGGGCCCATTACGGCCGACATGATAA	51	
pYES1L cloning vector F	CP-7	GCCCATAGCGATCGCCCTCGCCGCAGTTAATTAAAGTCA	39	Primer sets for amplifying vector for CPEC
pYES1L cloning vector R	CP-8	ATCGCATTAGGGCCCGCGGCGGTATCAGCGCGGCCGCCGCTGCGCTCGGTCGTTCGGGTTCTATATTA	68	

After successful transformation, 10 colonies were randomly picked for positive screening by junction PCR using fragment-specific primers (Table S1). After confirmation of correct size, positive colonies were seeded. To successfully perform *in vitro* transcription of the whole genomic length of 5′ cap-containing SARS-CoV-2 cDNA by T7 RNA polymerase, we linearized the assembled infectious clone plasmid by digestion at ApaI sites located up- and downstream of the insert (Fig. 1A). The recombinant SARS-CoV-2 virus was recovered by direct electroporation of the *in vitro*-transcribed, whole-genome-length RNA in BHK-21 cells. To further optimize the infectivity of RNA transcripts (25, 26), we further co-electroporated the SARS-CoV-2 N gene together with full-length SARS-CoV-2 RNA transcripts. Transfected BHK-21 cells were then overlaid onto Vero-E6 cells, and following transcription, the medium was replaced with Dulbecco's modified Eagle's medium (DMEM) containing 2% fetal bovine serum (FBS). After 72 to 96 h, transfected cells with cytopathic effects were observed, indicative of successful rescue of recombinant virus (WT-Rg); the resulting titer was estimated to be 7.8 × 10^5^ PFU/mL, and cells showed a plaque morphology similar to that of wild-type (WT-PC) (Fig. 1D). Furthermore, we observed comparable time courses and peaks of viral titers between regenerated SARS-CoV-2 (WT-Rg) and WT-PC SARS-CoV-2 (Fig. 1D). Collectively, these observations demonstrate successful recovery of SARS-CoV-2 virus using our CPEC method.

### Versatile primer design for point mutagenesis and the recovery of SARS-CoV-2 variants.

**FIG 2:**
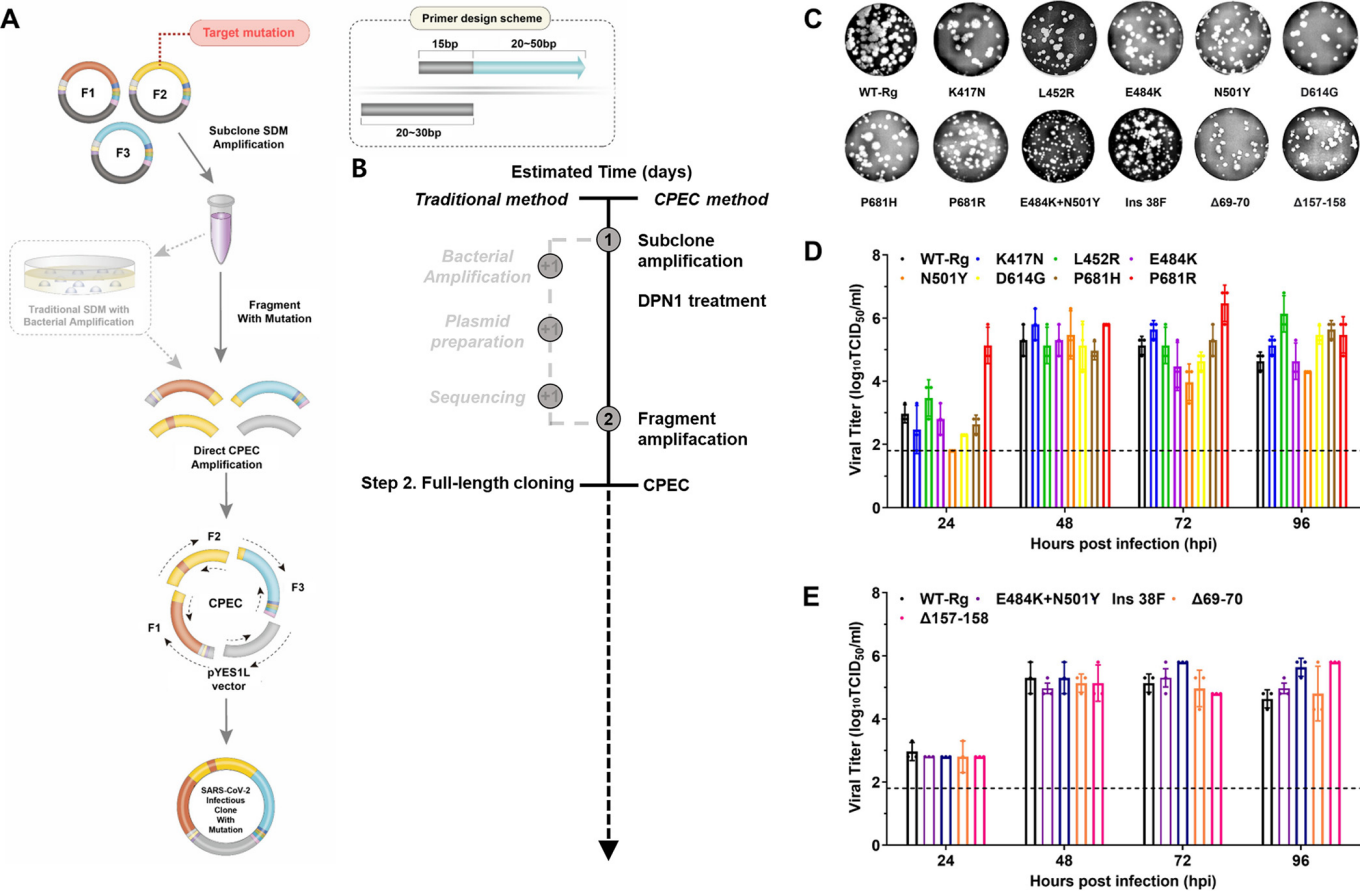
Single point mutagenesis for generation of SARS-CoV-2 variants. (A) Schematic diagram of the amplification scheme for simultaneous editing of point mutations. Utilizing the primer design scheme to amplify the target mutation of interest increases the usefulness and time efficiency of the method. (B) Time efficiency of the process. (C) Plaque formation by Rg-viruses containing point mutations. Each mutant characterized with respect to plaque size. (D) Viral growth kinetics of generated single point mutants. (E) Viral growth kinetics of generated adjacent double mutants and small-insertion and -deletion mutants. All mutants were assessed for growth capability for 96 h. Both plaque assays and viral growth experiments were performed in triplicate. Standard deviations were calculated and are indicated as error bars. Abbreviations: ins, insert; del, deletion.

**FIG 3:**
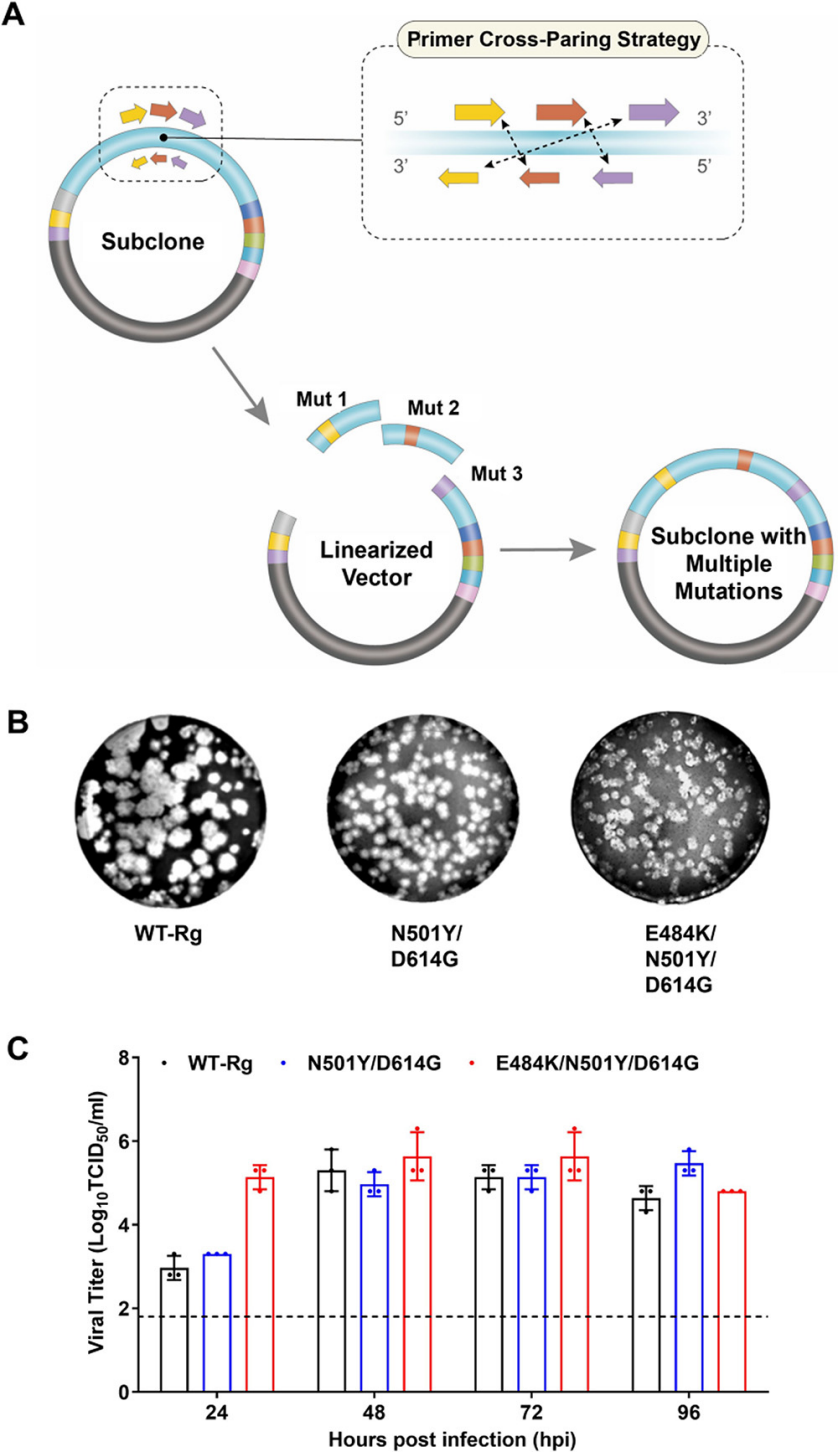
Multiple point mutagenesis for generation of SARS-CoV-2 variants. (A) Schematic diagram of cross-pairing amplification and in-fusion cloning in subclones for simultaneous editing of multiple point mutations. Utilizing cross-pairing primers to amplify the target mutation of interest increases the utility and time efficiency of the method. (B) Plaque formation. After rescue, each double and triple mutant was assessed with respect to its phenotypic characteristics. (C) *In vitro* growth of regenerated double and triple mutants. Both plaque assays and viral growth experiments were performed in triplicate. Data are presented as means ± SD (error bars). Abbreviations: ins, insert; del, deletion.

To further optimize the utility of the three-subclone/CPEC method, we demonstrated the ease of applying mutagenesis using a specific scheme for designing primers for both site-directed mutagenesis (SDM) (Fig. 2A) and in-fusion cloning methods for multiple mutagenesis (Fig. 3A). For this, a set of forward primers was designed containing 15 nt bases from the 5′ end that bind complementarily to the 15 bases from the 5′ end of the reverse primer and extend to 20 to 50 nt bases conferring the mutation of interest (Fig. 2A, dotted square). The SDM PCR product amplified using these primer sets was restriction digestion with DpnI and then used directly together with the respective CP-1 to -6 primer sets to amplify the mutated fragment for CPEC, obviating the need for bacterial transformation of the SDM product (Fig. 2A and B). Compared to traditional cloning methods, our CPEC mutagenesis method optimizes the process and reduces the time spent by up to 3 days (Fig. 2B). Using the resulting clones, we directly applied mutagenesis to generate mutants containing single substitutions (K417N, L452R, E484K, N501Y, D614G, P681H, and P681R) in the spike protein. We also introduced adjacent double substitutions (E484K+N501Y), small deletions (Δ69-70 and Δ157-158), and a small insertion (ins 38F) in NSP6 using a single primer set (Table 2). To verify the success of mutagenesis, we assessed the whole genome of all clones by Sanger sequencing using the custom-designed primers (Table S2). In addition, to verify the mutagenesis efficiency introduced by our specific primer design strategy, we confirmed positive mutant subclones by sequencing 10 colonies of each mutant subclone. This analysis revealed a mutagenesis efficiency of 30% to 100% (Table 3). To further determine the phenotypic characteristics of generated SARS-CoV-2 variants, we measured their replicative properties *in vitro* and compared them with the genetically reverse-generated WT-Rg. Each of the rescued recombinant SARS-CoV-2 variants exhibited cytopathic effect (CPE) and homogeneity of plaque size, as shown in Fig. 2C and Table 4. Notably, however, the plaque size of the combined E484K+N501Y variant was smaller than that of other variants. Most rescued mutagenic viruses possessed titers in the range of 2.0 × 10^4^ to 4.9 × 10^5^ PFU/mL compared with 7.2 × 10^5^ PFU/mL and 7.8 × 10^5^ PFU/mL for isolated WT-PC and WT-Rg, respectively (Fig. 2C; Table 4). The replication kinetics of single mutants revealed that a peak was reached within 48 h in Vero E6 cells for almost all tested mutants (Fig. 2C). Notably, all mutants showed an infectious titer similar to that of control WT-Rg (log_10_ TCID_50_/mL = 5.134 ± 0.236) after 48 h. Nonetheless, the efficiency and rapidness of three-subclone/CPEC combined with the introduced primer design scheme support the utility of this approach in performing SARS-CoV-2 point mutagenesis. The genetic correspondence of single-passaged WT recombinant viruses to their infectious clones was verified by deep sequencing, which showed no unwanted mutations (Table 4).

**TABLE 2:** Primers for SARS-CoV-2 mutagenesis

Target gene	Primer name	Binding site	Sequence (5′ to 3′)^*a*^	Base pair
For single or multiple point mutagenesis				
Spike	Point Mutation K417N F	22,798 to 22,832	AGGGCAAACTGGAAA**t**ATTGCTGATTATAATTATA	35
	Point Mutation K417N R	22,792 to 22,812	TTTCCAGTTTGCCCTGGAGCG	21
	Point Mutation L452R F	22,898 to 22,937	GGTGGTAATTATAATTACC**g**GTATAGATTGTTTAGGAAGT	34
	Point Mutation L452R R	22,893 to 22,912	ATTATAATTACCACCAACCT	24
	Point Mutation E484K F	22,997 to 23,030	CCTTGTAATGGTGTT**a**AAGGTTTTAATTGTTACT	34
	Point Mutation E484K R	22,988 to 23,011	AACACCATTACAAGGTGTGCTACC	24
	Point Mutation N501Y F	23,048 to 23,079	GGTTTCCAACCCACT**t**ATGGTGTTGGTTACCA	32
	Point Mutation N501Y R	23,038 to 23,062	AGTGGGTTGGAAACCATATGATTGT	25
	Point Mutation D614G F	23,388 to 23,418	CTGTTCTTTATCAGG**g**TGTTAACTGCACAGA	31
	Point Mutation D614G R	23,377 to 23,402	CCTGATAAAGAACAGCAACCTGGTTA	26
	Point Mutation P681H F	23,589 to 23,622	CTCAGACTAATTCTC**a**TCGGCGGGCACGTAGTGT	34
	Point Mutation P681H R	23,582 to 23,603	GAGAATTAGTCTGAGTCTGATA	22
	Point Mutation P681R F	23,589 to 23,623	CTCAGACTAATTCTC**g**TCGGCGGGCACGTAGTGTA	35
	Point Mutation P681R R	23,581 to 23,603	GAGAATTAGTCTGAGTCTGATAA	23
	Multi PM E484K-N501Y F	22,997 to 23,068	CCTTGTAATGGTGTT**a**AAGGTTTTAATTGTTACTTTCCTTTACAATCATATGGTTTCCAACCCACT**t**ATGGT	72
	Multi PM E484K-N501Y R	22,988 to 23,011	AACACCATTACAAGGTGTGCTACC	24
For insertion or truncation mutagenesis				
ORF1a	ORF1a 11085 TTT F	11,068 to 11,103	TTTGTTCTTTTTTTT**cttc**TATGAAAATGCCTTTTTACC	39
	ORF1a 11085 TTT R	11,051 to 11,080	AAAAAAGAACAAAGACCATTGAGTACTCTG	30
Spike	69/70 Deletion F	21,752 to 21,793	TGGTTCCATGCTATATCTGGGACCAATGGTACTAAG	36
	69/70 Deletion R	21,743 to 21,766	TATAGCATGGAACCAAGTAACATT	24
	157/158 Deletion F	22,014 to 22,056	GTTGGATGGAAAGTGGAGTTTATTCTAGTGCGAATAA	37
	157/158 Deletion R	22,007 to 22,028	CACTTTCCATCCAACTTTTGTT	22
ORF7A	GFP Insert F	Turbo GFP	GAGAGCGACGAGAGCGGCCT	20
	GFP Insert R	Turbo GFP	ATTCTTCACCGGCATCTGCA	20
	GFP Vector F	27,677 to 27,700	ATGCCGGTGAAGAATAACTTTACTCTCCAATTTTTCTTA	39
	GFP Vector R	27,409 to 27,432	GCTCTCGTCGCTCTCAGCGAGTGTTATCAGTGCCAAGAA	39
	7A-Truncation F	27,677 to 27,711	AACTTTACTCTCCAATTTTTCTTATTGTTGCGGCA	35
	7A-Truncation R	27,412 to 27,432	TTGGAGAGTAAAGTTAGCGAGTGTTATCAGTGCCAA	36

a Bold small letters indicate the substitution or insertion in the primer sequence.

**TABLE 3:** Mutagenesis efficiency introduced by a specific primer design scheme in subclone

Mutagenesis	Mutation	Target gene (subclone)	Colonies picked/total colonies	Mutagenesis efficiency (%)^*a*^
Single to multiple point mutations^*b*^^,^^*c*^	WT-Rg Marker-1 (A7486T/T7489A)	ORF1ab (F1)	10/94	100%
	K417N	Spike (F3)	10/56	90%
	L452R	Spike (F3)	10/64	60%
	E484K	Spike (F3)	10/38	70%
	N501Y	Spike (F3)	10/42	90%
	D614G	Spike (F3)	10/67	60%
	P681H	Spike (F3)	10/52	70%
	P681R	Spike (F3)	10/94	30%
	E484K+N501Y	Spike (F3)	10/36	50%
	Ins 38F	Nsp6 (F2)	10/46	100%
	Δ69-70	Spike (F3)	10/52	60%
	Δ157-158	Spike (F3)	10/61	70%
Multifragment point mutations^*b*^	N501Y+D614G	Spike (F3)	10/32	90%
	E484K+N501Y+D614G	Spike (F3)	10/38	80%
Large deletion^*d*^	ΔORF7A	ORF7A (F3)	10/82	50%
Large insertion^*d*^	GFP	ORF7A (F3)	10/62	100%

a Mutagenic efficiency is defined as the percentage of positive mutated subclones among selected clones.

b Mutagenesis was inferred from the recent publications of Li et al. (2020).

c Khalid et al. (2021).

d Thi Nhu Tao et al. (2020).

**TABLE 4:** Phenotypic and genomic characterization of recovered SARS-CoV-2 recombinant viruses

Sample name^*c*^	CPE	Plaque assay (PFU/mL)	Mean plaque size ± SD (mm)	Mutation of virus after single passage				
				Nucleotide position	Region	Nucleotide change	Amino acid change	Proportion (%)
WT-PC	Yes	7.2 × 10^5^	1.90 ± 0.62	−	−	−	−	−
WT-Rg	Yes	7.8 × 10^5^	1.92 ± 0.66	−	−	−	−	−
K417N-Rg	Yes	2.5 × 10^5^	2.30 ± 0.53	19,392	ORF1ab	A→C	−	27.12
				27,372	ORF6	A→C	−	48.29
L452R-Rg	Yes	5.3 × 10^4^	1.39 ± 0.56	−	−	−	−	−
E484K-Rg	Yes	3.8 × 10^5^	1.89 ± 0.50	−	−	−	−	−
N501Y-Rg	Yes	5.5 × 10^4^	1.63 ± 0.33		−	−	−	−
D614G-Rg	Yes	2.2 × 10^5^	2.13 ± 0.38	12,124	ORF1ab	A→C	−	48.26
				17,748	ORF1ab	T→C	−	33.82
				19,696	ORF1ab	A→C	I6478L	45.29
P681H-Rg	Yes	4.2 × 10^4^	1.97 ± 0.72		−	−	−	−
P681R-Rg	Yes	4.9 × 10^5^	1.56 ± 0.65	23,606	Spike	C→T	R682W^*a*^	53.72
E484K+N501Y-Rg	Yes	1.5 × 10^5^	1.23 ± 0.41	1,180	ORF1ab	A→C		97.71
Ins 38F-Rg	Yes	2.2 × 10^5^	1.55 ± 0.46	−	−	−	−	−
Δ69-70-Rg	Yes	3.1 × 10^5^	1.71 ± 0.47	20,553	ORF1ab	A→G		37.61
Δ157-158-Rg	Yes	6.9 × 10^4^	1.42 ± 0.65	22,097	Spike	C→T	L179F^*b*^	44.92
N501Y+D614G-Rg	Yes	2.0 × 10^4^	1.52 ± 0.36	−	−	−	−	−
E484K+N501Y+D614G-Rg	Yes	2.6 × 10^5^	1.40 ± 0.25	−	−	−	−	−
ΔORF7A-Rg	Yes	2.1 × 10^4^	1.36 ± 0.27	808	ORF1ab	A→T		99.74
GFP-Rg	Yes	6.1 × 10^4^	0.88 ± 0.20	708	ORF1ab	A→T	E148V	47.72

a R682W mutation in spike region was inferred from the publications of Escalera et al. (27).

b L179F mutation in spike region was inferred from the publications of Laha et al. (28).

c WT, wild-type; Rg, reverse genetics; CPE, cytopathic effect; PFU, plaque forming units; SD, standard deviation.

### Versatility of SDM primers for the simultaneous editing of multiple mutations and generation of corresponding variants.

To edit DNA plasmids containing multiple mutations that are relatively distinct from each other, we further determined whether the primers designed for point mutagenesis were versatile enough for use in simultaneous introduction of multiple point mutations (Fig. 3A). For substitution of N501Y and D614G in the spike-encoding gene, we applied the same scheme for primer designs. However, during generation of the insert containing the targeted mutation, the forward primers cross-pair with the reverse primers of the adjacent primer set; specifically, the forward primer of the N501Y cross-pairs with the reverse primer of D614G, whereas the forward primer used to generate the insert containing D614G cross-pairs with the reverse primer of N501Y. This cross-pairing primer amplification scheme generated fragments that shared 15 homologous overlapping bases on their ends. Each amplified PCR product contains overlapping ends homologous to those of the vector or adjacent fragments that serve to facilitate subsequent in-fusion reactions. Once the subcloned fragment is successfully transformed with multiple mutations, the plasmid can be assembled and cloned to produce a full-length genome by CPEC. Applying this same design scheme, we generated SARS-CoV-2 variants with a triple E484K/N501Y/D614G mutation. The genetic correspondence of each single-passaged recombinant mutant virus to its infectious clone was also verified by deep sequencing. Among the 16 recombinant viruses, 50%, including K417N-Rg, D614G-Rg, P681R-Rg, E484K+N501Y-Rg, Δ69-70-Rg, Δ157-158-Rg, ΔORF7A-Rg, and GFP-Rg, acquired one to three unwanted nucleotide mutations (Table 4). Most of these mutations were synonymous, but in four cases—D614G-Rg, P681R-Rg, Δ157-158-Rg, and GFP-Rg—the virus acquired one amino acid substitution. The proportion of most mutations was in the 27% to 54% range, although two synonymous mutations acquired in 484K+N501Y-Rg and ΔORF7A-Rg viruses accounted for more than 97% of rescued and single-passaged isolates (Table 4). No nucleotide mutations were found in the *in vitro*-transcribed viral RNA genome before cell transfection (data not shown). Overall, more than 70% of mutations showed a proportion less than 50%, and some of the amino acid changes (i.e., R682W and L179F in the spike protein) are reported to be adaptive mutations in a cell or human infection (27, 28). Therefore, these mutations were potentially acquired during virus propagation in cells.

Observations of successfully regenerated mutants showed that plaque sizes of recombinants containing double or triple mutations in the spike were smaller than those of WT-Rg (Fig. 3B; Table 4). However, the growth kinetics of the E484K/N501Y/D614G triple mutant in Vero E6 cells (mean peak log_10_ TCID_50_/mL value = 5.468 ± 0.236) revealed that viral titers of this mutant were higher than those of the WT-Rg SARS-CoV-2 virus (mean peak log_10_ TCID_50_/mL value = 5.134 ± 0.236). Overall, the use of cross-pairing primers and a two-step amplification scheme for subclones reduces the complexity of the method for generating multiple point mutations in SARS-CoV-2 while increasing time efficiency.

### Mutagenesis for introducing a large insertion and deletion in the SARS-CoV-2 genome.

**FIG 4:**
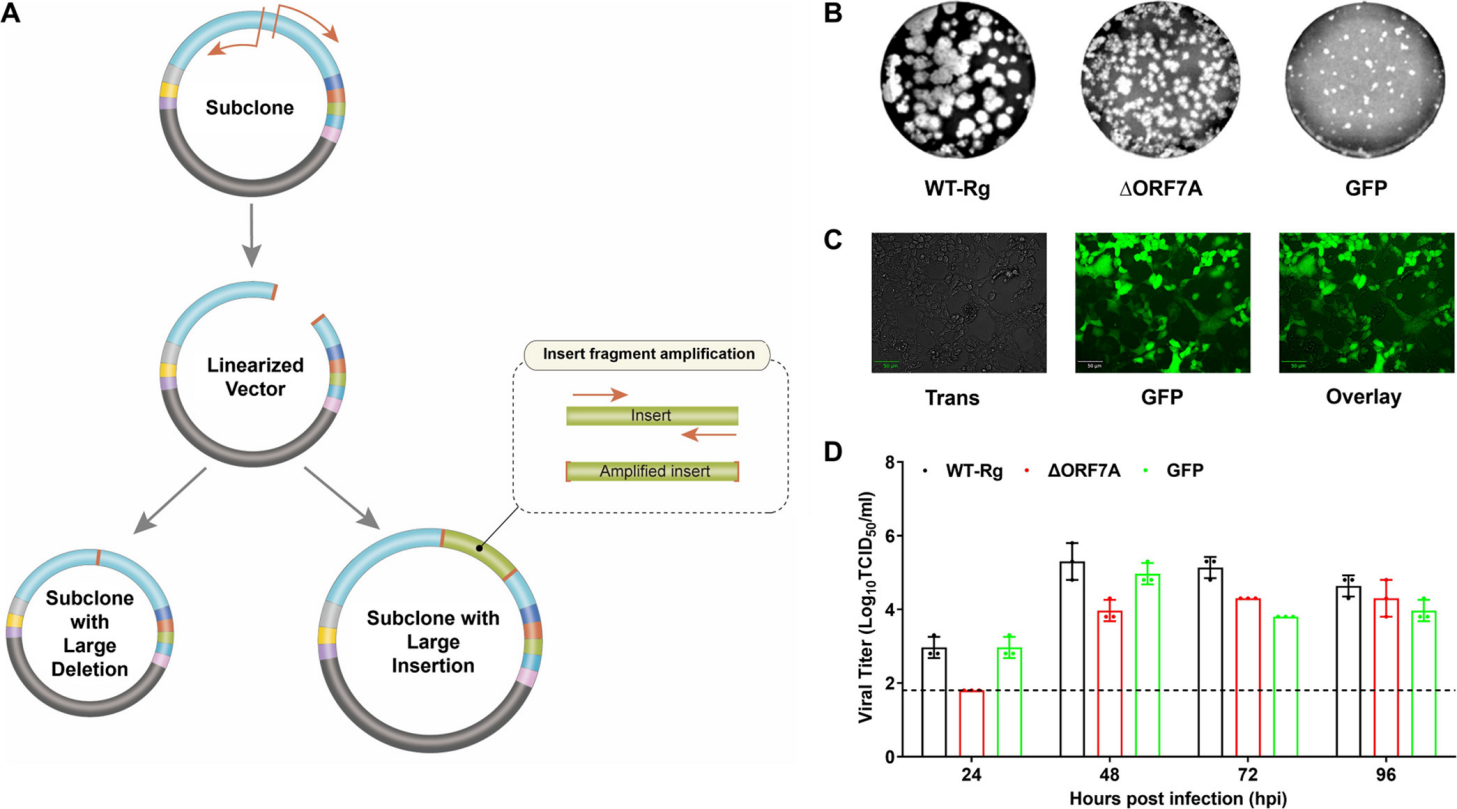
Mutagenesis strategy for generation of SARS-CoV-2 variants with large insertions or deletions. (A) Schematic diagram of the method for performing large-scale mutagenesis. Large insertions or deletions are processed by PCR, followed by in-fusion cloning. (B) Plaque formation by rescued viruses containing large-scale edits. (C) Image showing GFP fluorescence by a GFP gene-expressing SARS-CoV-2 variant. (D) *In vitro* growth kinetics of SARS-CoV-2 viruses with a large deletion or insertion.

To determine whether our primer design scheme can also be applied to large-scale editing of the SARS-CoV-2 genome, we recovered a gene-deleted SARS-CoV-2 Rg-virus after removal of the ORF7A gene (27,410 to 27,710 nt), a process that eliminated a total of 244 bases or 81 amino acids (Fig. 4A). For truncation of more than 30 nt, a two-step amplification is recommended, with an initial step in which a fragment containing the deletion is generated by PCR in a subclone followed by CPEC for whole-genome cloning. We successfully rescued a SARS-CoV-2 variant containing a large deletion (ΔORF7A) and noted that the recombinant virus exhibited a lower viral titer and reduced plaque formation compared with WT-Rg virus (Fig. 4B; Table 4).

We also explored whether the same primer design scheme could be used to generate a large insertion in the SARS-CoV-2 genome. In this case, the ORF7A gene was deleted and replaced with Turbo GFP, a total of 694 nucleotides (nt), using two designed primer sets, one for the GFP insert and one for the vector component (Table 4). To subclone GFP-mutant F3, we in-fused and transformed the two amplified fragments (Fig. 4A) and used CPEC to process the GFP-mutant F3 with the other fragments. GFP-expressing SARS-CoV-2 formed smaller plaques relative to those of WT-Rg virus (Fig. 4B). Notably, cells containing GFP were readily observed from 48 to 72 h postinfection, indicating the successful recovery of infectious virus with GFP expression (Fig. 4C). Although the replication kinetics of the recovered GFP-expressing viruses were slightly lower than those of the WT-Rg strain (Fig. 4D), the SARS-CoV-2 clone expressing GFP will aid in future antiviral screening and quantification assays.

## DISCUSSION

The public availability of complete genomes has enabled researchers to monitor changes in the viral genome of SARS-CoV-2 since the first emergence of its variants in 2020. Several mutations that have occurred in the genetic composition of SARS-CoV-2 have enhanced the fitness and transmissibility of the virus, making it more difficult to control (29–31). Principle concerns have been the vast array of point mutations in the S protein, which primarily aids in the entry of the virus into the host cell (11, 13, 25, 32). The presence of these mutations may reduce the efficacy of available vaccines as they weaken binding of the antibody (32). Because the combination of these point mutations has given rise to current SARS-CoV-2 VOCs and VOIs, which in turn impact public health, there is an urgent need for the development of methods for determining the impacts of these mutations on viral infectivity and antigenicity.

Among RNA viruses, coronaviruses have the largest genome size, which reaches almost 30 kb (6). Hence, generating an infectious clone has always been a challenge. Compared to a previously reported reverse genetics method (14, 16, 19, 30), the method utilized in the current study employed fewer fragments (three versus seven to 10) to facilitate various mutagenesis strategies in subclones and mitigate errors by reducing CPEC PCR cycles (24). Although previously utilized yeast cloning and other traditional cloning methods show proven cloning efficiency and are unhampered by the large genome size of SARS-CoV-2, they require considerable time for successful completion of the assembly process. Hence, in this study, we demonstrated how the application of CPEC simplified the process and reduced the cloning time required for the most available method for recovering SARS-CoV-2. The advantages of the CPEC method rely on the overlapping strands of the fragments and cloning vectors during the amplification and the quality of the PCR polymerase used. Here, we specifically designed primers that generate fragments and a vector with double-overlapping homologous termini. We also used GXL DNA polymerase, which allows successful amplification of products more than 40 kb in length, like SARS-CoV-2 genome, together with a 10-kb vector. Compared with transformation-associated recombination (TAR) cloning in yeast, CPEC can substantially reduce the number of steps as well as the time required for assembly. Thus, compared with previous reverse genetics systems developed, this customized CPEC approach shows improved efficiency, speed, and simplicity in regenerating large-size genome viruses like SARS-CoV-2.

We also demonstrate an easier and uncomplicated guide for performing mutagenesis in the SARS-CoV-2 genome for amplification of fragments. The versatility of our designed primers has expanded the utility of each primer, reducing the cost and time needed for redesigning additional primers when performing multiple-substitution mutagenesis. The primer design scheme for mutagenesis devised in this study can be readily applied for use in SDM and in-fusion cloning methods, facilitating simple and broad mutagenesis, as demonstrated here.

Notably, VOCs that have arisen contain the following key mutations in the spike protein: N501Y and D614G, common among all; E484K and L452R, common among Alpha VOC (B.1.1.7); K417N for Delta (B.1.617.2); and P681H for Gamma (P.1) (7, 12). Additional contributions of VOCs include observed deletions in the spike (Δ69/70) (25) and a 382-nt deletion in ORF8 in an isolate from a patient in Taiwan (33). In the current study, we found that most single point mutations minimally impacted the growth properties of SARS-CoV-2, whereas a triple mutant significantly improved *in vitro* viral growth compared with that of WT-Rg. The results of our phenotypic assays potentially support the interpretation that combinations of each of these mutations in the spike have a greater impact on the replicability of the virus, and that the combination of these mutagenic events has allowed SARS-CoV-2 to evolve detrimental changes in pathogenicity and epidemiology (7). Recombinants generated using the approach described here can be utilized in studying emerging variants, especially studies designed to assess the impact of these variants on antibody therapies. In addition, SARS-CoV-2 viruses with large deletions of ORF7A and insertion of GFP, successfully and simply generated via this method, will contribute to the development of live-attenuated vaccines or strategies for screening antivirals. Overall, our study enables a simple, uncomplicated approach for performing mutagenesis and recovering SARS-CoV-2 variants for further study and diagnostic evaluation, providing a rapid preparedness capability in the face of continuously emerging VOCs.

## MATERIALS AND METHODS

### Cells and virus.

Vero E6 (ATCC, CRL1586) and Baby Hamster Kidney-21 (BHK-21) cell lines utilized in this study were obtained from Korean Cell Line Bank (KCLB; Seoul, Republic of Korea). Vero E6 cells were maintained in DMEM (Gibco-Invitrogen, Carlsbad, CA, USA), whereas BHK-21 cells were maintained in minimum essential medium-alpha (MEM-α; Gibco-Invitrogen), both supplemented with 5% to 10% (vol/vol) heat-inactivated FBS and 1% antibiotics (Gibco-Invitrogen) at 37°C and 5% CO_2_. The SARS-CoV-2 virus resource (NCCP43326) for this study was provided by the National Culture Collection for Pathogens, Korea National Institute of Health, and was propagated at 37°C and 5% CO_2_ in Vero E6 cells in 2% FBS containing DMEM. After 72 to 96 h, viral supernatants were harvested and processed for storage at −80°C before use.

### Primers and viral cDNA synthesis.

The KDCA (BetaCoV/Korea/KCDC03/2020 [GISAID id: EPI-ISL: 407193]) strain, from the 5′-untranslated region (UTR) to 3‘-UTR (29,870 bp), was used as a reference strain. For the design of primer sets (Table 1), the KDCA sequence together with all available SARS-CoV-2 sequences in the GenBank database from January 2020 to November 2021 were aligned using CLC Genomics Workbench 7.0 (Qiagen, Hilden, Germany). SARS-CoV-2 genomic RNA was extracted using a RNeasy minikit (Qiagen, Hilden, Germany) following the manufacturer’s instructions. Viral cDNA was synthesized using reverse transcription (RT) primers (RT-1, RT-2, and RT-3) (Table 1) at 65°C for 5 min. After a 2-min incubation on ice, Superscript III Reverse Transcriptase (Invitrogen, Waltham, MA, USA) was added and the elongation process was allowed to proceed at 50°C for 90 min, followed by inactivation of the RT enzyme at 70°C for 15 min.

### Subcloning of the SARS-CoV-2 genome.

The SARS-CoV-2 genome was subcloned by first dividing it into three fragments corresponding to approximately 8.7 to 11.8 kb regions (Fig. 1A) by digesting the vector with AgeI, which serves to prevent back-transformation, followed by amplification of fragments using Phusion High Fidelity DNA polymerase (New England Biolabs, Ipswich, MA, USA) with specifically designed primer sets: Fragment 1, SP-1 and SP-2; Fragment 2, SP-3 and SP-4; and Fragment 3, SP-5 and SP-6; the final subcloning vector was amplified using primers SP-7 and SP-8 (Table 1; Fig. S2A). All fragments overlapped each other by at least 30 bp, with F1 and F2 overlapping by 450 bp. After amplification of the vector, back-transformation was further prevented by incubating the PCR product with DpnI to digest the methylated plasmids used as template. PCR was performed using an initial denaturation step of 98°C for 30 s, followed by 34 cycles of denaturation at 98°C for 10 s, annealing at 68 to 69°C for 30 s, and extension at 72°C for 5 min and 30 s for inserts and 2 min for the vector, with a final extension step of 72°C for 10 min. Electrophoretic analyses of PCR products for each fragment and vector showed bands of 8.7 to 11.8 kb for fragments F1, F2, and F3, and 3.16 kb for the vector. The amplified PCR products were purified using a NucleoSpin Gel and PCR Clean-up kit (Macherey-Nagel, Hoerdt, France), and then each fragment was cloned into the subcloning vector using an In-Fusion HD cloning kit (TaKaRa Bio Inc., Kusatsu, Shiga, Japan), following the manufacturer’s protocol. The in-fused clone samples were transformed into OneShot TOP10 chemically competent cells (Invitrogen) using a heat shock protocol and incubated on ampicillin-containing Luria Bertani (LB) plates at 25°C for 48 h. Positive clones were selected by performing colony PCR with enzyme 2× TOPsimple DyeMIX-tenuto (Enzynomics Co., Yuseong-gu, Daejeon, Republic of Korea), which detects junctions of each fragment using custom-designed primers (Fig. S2B; Table S1). The PCR conditions used were an initial denaturation step of 95°C for 30 s, followed by 34 cycles of denaturation at 95°C for 10 s, annealing at 58°C for 90 s, and extension at 72°C for 90 s, with a final extension step of 72°C for 2 min. Also, two silent mutations, A7486T and T7489A, were introduced in F1 subclone to confirm whether rescued viruses from transfected RNA or not.

### CPEC using vector and three SARS-CoV-2 genome fragments.

Each subclone was amplified individually using its respective CPEC primers (Primer code: CP-1 to CP-6) (Table 1) under the following conditions: initial denaturation step of 98°C at 30 s, followed by 34 cycles of denaturation at 98°C for 10 s, annealing at 69°C for 30 s, and extension at 72°C for 5 min and 30 s, with a final extension step of 72°C for 10 min. The vector, pYES1L, was then also amplified under the same conditions using the designed primer set CP-7/CP-8 (see Table 1). All amplified products were treated with DpnI (New England Biolabs) for up to 8 h, and purified using a Macherey-Nagel NucleoSpin Gel and PCR Clean-up kit (Hoerdt). Inserts (F1, F2, and F3) and pYES1L vector were assembled through the CPEC method using 0.1 pmol of each fragment. CPEC processing employed PrimeSTAR GXL DNA polymerase (TaKaRa Bio Inc.) under the following conditions: an initial denaturation step of 98°C for 2 min, followed by 15 cycles of denaturation at 98°C for 10 s, annealing at 55°C for 15 s, and extension at 68°C for 15 min, with a final extension step of 68°C for 15 min. The CPEC product was electroporated into electrocompetent cells (Invitrogen) following the manufacturer’s instructions and incubated on an LB agar plate supplemented with a 0.01% (wt/vol) spectinomycin for 24 to 36 h at 37°C. For confirmation of recombinant clones, colony PCR targeting junctions of each fragment was performed using specifically designed CPEC junction primers (Fig. S2C; Table S1) with 2× TOPsimple DyeMIX Tenuto (Enzynomics Co.) and the following PCR conditions: an initial denaturation step at 95°C for 2 min, followed by 34 cycles of 95°C for 30 s, 61°C for 30 s, and 72°C for 1 min and 30 s, with a final extension step of 72°C for 5 min.

### SARS-CoV-2 mutagenesis of subclones and direct construction of a full-length infectious clone variant.

The SARS-CoV-2 genome was manipulated by applying mutagenesis strategies to the constructed subclone plasmids. Noted variations in the SARS-CoV-2 genome were selected and introduced into the corresponding target fragments using designed, versatile primer sets. Overlapping nucleotides were positioned 15 nt from the 5′ end in forward primers, which contain a further 20 to 50 nt extension that confers the mutation of interest. The reverse primer contained 15 bases of the paired forward primer’s 5′ end in reverse complementary orientation and extended a further 20 to 30 bases (Fig. 2A; Table 2). In this study, we designed a versatile primer scheme for generating single or multiple point mutations, a large insertion, and a large truncation in spike and ORF7A genes of SARS-CoV-2, as shown in Table 2. To introduce point mutations or adjacent double mutations, we utilized SDM PCR of target subclones employing PrimeSTAR GXL DNA polymerase (TaKaRa Bio Inc.). The cycling conditions for PCR mutagenesis were as follows: an initial denaturation step of 98°C for 2 min, followed by 15 cycles of denaturation at 98°C for 10 s, annealing at 55 to 60°C for 15 s, and extension at 68°C for 15 min, with a final extension step of 68°C for 15 min.

The resulting PCR products produced through mutagenesis can be generated as subclones by transformation or undergo PCR and CPEC for use in constructing a full-length clone. In the former case, PCR products can be used for transformation after DpnI treatment to generate subclones possessing target mutation(s); in the latter, the site-directed mutagenesis PCR product can be used as a template to directly generate a fragment for CPEC by using the appropriate primer sets (CP-1 to CP-6) (Table 1). The cycling conditions were as follows: an initial denaturation step of 98°C for 2 min, followed by 15 cycles of denaturation at 98°C for 10 s, annealing at 55 to 60°C for 15 s, and extension at 68°C for 15 min, with a final extension step of 68°C for 15 min. The composition of the PCR mixture followed the standard protocol of the manufacturer.

### *In vitro* transcription.

The SARS-CoV-2 whole genome was first separated from clones using the restriction enzyme ApaI and then purified using a manual phenol-precipitation method (34). The linearized plasmid was processed for *in vitro* transcription using a mMessage mMachine T7 Transcription kit (Thermo Fisher Scientific, Waltham, MA, USA) following the manufacturer’s protocol, with slight modifications. Briefly, a 40-μL reaction containing 6 μL GTP (GTP:cap ratio, 1:1) and 12 μg of DNA template was incubated for 8 to 12 h at 32°C, after which template DNA was removed by adding 2 μL of Turbo DNase and incubating for 15 min at 37°C. RNA was subsequently extracted by lithium chloride precipitation. The stability of viral RNA during replication in transfected cells was improved by also transcribing the N gene of SARS-CoV-2 *in vitro* and co-transfecting it into the cell. The N gene DNA template was prepared by PCR as previously described (14). Transcription of the SARS-CoV-2 N gene was performed by incubating with 1 μL of GTP (GTP: cap ratio, 1:1) in a 20-μL reaction at 37°C for 3 h.

### Virus recovery.

SARS-CoV-2 RNA transcripts were transfected into BHK-21 cells by electroporation as previously described (14). Briefly, 10 μg of whole-genome transcripts, 2 μg of N gene transcript, and 8 × 10^6^ BHK cells in 800 μL of Opti-MEM were mixed in a 4-mm gap cuvette and incubated for 10 min on ice. After incubation, cells were electroporated (voltage, 850V; pulse, 5 times with 0.01-ms pulse length and 1-s pulse interval) using a BTX Gemini X2 twin-wave electroporator and Opti-MEM as an electroporation reagent. After a 5-min recovery, electroporated cells were seeded on a monolayer of Vero E6 cells in a T-75 flask and incubated with 10 mL of 5% DMEM at 37°C and 5% CO_2_. After 24 h, the medium was replaced with 2% DMEM and plates were further incubated for a minimum of 72 h or until ~90% of cells exhibited virus-mediated cytopathic effects. The rescued virus—Passage 0 (P0)—was inoculated into another T-75 flask containing Vero E6 cells and passaged. After incubating for 72 to 96 h at 37°C and 5% CO_2_, cell supernatants (P1) were harvested. After virus recovery, viral RNA was sequenced to confirm it has silent mutations (A7486T and T7489A) by the Sanger sequencing method for ensuring the viruses were rescued from transfected RNA (Fig. S3). To confirm whether viruses have undesired mutations, viral RNAs were extracted from single-passaged viruses using RNeasy minikit (Qiagen, Hilden, Germany), sequenced by deep sequencing method (DNALINK, Republic of Korea) and analyzed by CLC genomics workbench (Qiagen, Hilden, Germany).

### Virus quantification and plaque formation.

Plaque formation and the concentration of recombinant viral stocks relative to their parental viruses were determined by preparing Vero E6 cells in 6-well plates and infecting with 1 mL of 10-fold serially diluted recombinant/parental viruses. Plates were incubated at 37°C and 5% CO_2_ for 1 h to allow for virus–cell attachment. During this incubation period, a 0.7% DMEM-agarose overlay medium was prepared by mixing 1.4% agarose and 2× DMEM media. After incubating, the viral inoculum in each well was removed and replaced with a 3-mL volume of overlay medium. The plate with overlay was then cooled at 4°C for 10 min before incubating in a 37°C and 5% CO_2_ incubator. At 96 h postinfection, the cells were fixed with 10% formaldehyde and stained with 1% (wt/vol) crystal violet. Wells containing 20 to 100 plaques were selected and analyzed using ImageJ software (35) (National Institute of Health, Bethesda, Maryland), and the titer of virus stocks, in PFU, was calculated.

### *In vitro* growth kinetics.

For comparison of *in vitro* viral growth kinetics, cells were infected with wild-type or recombinant SARS-CoV-2, diluted to a multiplicity of infection (MOI) of 0.0001. All plates were incubated at 37°C and 5% CO_2_ for 1 h. After the viral adsorption period, media were replaced with DMEM containing 2% (vol/vol) FBS. Each cell culture supernatant was harvested at 24, 48, 72, and 96 h postinfection, and viral titers at each time point were measured as TCID_50_ (median tissue culture infectious dose), calculated based on the Reed-Muench method.

## References

[1] World Health Organization. 2021. COVID-19 weekly epidemiological update - 19–27 July 2021. World Health Organization.

[2] Blackstone NW, Blackstone SR, Berg AT. 2020. Variation and multilevel selection of SARS-CoV-2. Evolution 74:2429–2434. doi:10.1111/evo.14080. 32880957 PMC7461403

[3] Claro IM, da Silva Sales FC, Ramundo MS, Candido DS, Silva CAM, de Jesus JG, Manuli ER, de Oliveira CM, Scarpelli L, Campana G, Pybus OG, Sabino EC, Faria NR, Levi JE. 2021. Local transmission of SARS-CoV-2 Lineage B.1.1.7, Brazil, December 2020. Emerg Infect Dis 27:970–972. doi:10.3201/eid2703.210038. 33496249 PMC7920684

[4] Cyranoski D. 2021. Alarming COVID variants show vital role of genomic surveillance. Nature 589:337–338. doi:10.1038/d41586-021-00065-4. 33452508

[5] Sanjuán R, Domingo-Calap P. 2016. Mechanisms of viral mutation. Cell Mol Life Sci 73:4433–4448. doi:10.1007/s00018-016-2299-6. 27392606 PMC5075021

[6] Su S, Wong G, Shi W, Liu J, Lai ACK, Zhou J, Liu W, Bi Y, Gao GF. 2016. Epidemiology, genetic recombination, and pathogenesis of coronaviruses. Trends Microbiol 24:490–502. doi:10.1016/j.tim.2016.03.003. 27012512 PMC7125511

[7] World Health Organization. 2021. Tracking SARS-CoV-2 variants. https://www.who.int/en/activities/tracking-SARS-CoV-2-variants/. Accessed 26 June 2021.

[8] Erol A. 2021. Are the emerging SARS-COV-2 mutations friend or foe? Immunol Lett 230:63–64. doi:10.1016/j.imlet.2020.12.014. 33400966 PMC7832648

[9] Burki T. 2021. Understanding variants of SARS-CoV-2. Lancet 397:462. doi:10.1016/S0140-6736(21)00298-1. 33549181 PMC7906644

[10] Public Health England. 2020. Investigation of novel SARS-COV-2 variant: variant of concern 202012/01. Public Health England, London, United Kingdom. https://assets.publishing.service.gov.uk/government/uploads/system/uploads/attachment_data/file/947048/Technical_Briefing_VOC_SH_NJL2_SH2.pdf.

[11] Danchin A, Timmis K. 2020. SARS-CoV-2 variants: relevance for symptom granularity, epidemiology, immunity (herd, vaccines), virus origin and containment? Environ Microbiol 22:2001–2006. doi:10.1111/1462-2920.15053. 32367648 PMC7267449

[12] Chen J, Gao K, Wang R, Wei GW. 2021. Revealing the threat of emerging SARS-CoV-2 mutations to antibody therapies. J Mol Biol 433:167155. doi:10.1016/j.jmb.2021.167155. 34273397 PMC8277955

[13] Crits-Christoph A, Kantor RS, Olm MR, Whitney ON, Al-Shayeb B, Lou YC, Flamholz A, Kennedy LC, Greenwald H, Hinkle A, Hetzel J, Spitzer S, Koble J, Tan A, Hyde F, Schroth G, Kuersten S, Banfield JF, Nelson KL. 2021. Genome sequencing of sewage detects regionally prevalent SARS-CoV-2 variants. mBio 12. doi:10.1128/mBio.02703-20. PMC7845645 33468686

[14] Xie X, Muruato A, Lokugamage KG, Narayanan K, Zhang X, Zou J, Liu J, Schindewolf C, Bopp NE, Aguilar PV, Plante KS, Weaver SC, Makino S, LeDuc JW, Menachery VD, Shi P-Y. 2020. An infectious cDNA Clone of SARS-CoV-2. Cell Host Microbe 27:841–848.e3. doi:10.1016/j.chom.2020.04.004. 32289263 PMC7153529

[15] Liu X, Zaid A, Freitas JR, McMillan NA, Mahalingam S, Taylor A. 2021. Infectious clones produce SARS-CoV-2 that causes severe pulmonary disease in infected K18-human ACE2 mice. mBio 12:e00819-21. doi:10.1128/mBio.00819-21. 33879586 PMC8092263

[16] Ye C, Chiem K, Park JG, Oladunni F, Platt RN, 2nd, Anderson T, Almazan F, de la Torre JC, Martinez-Sobrido L. 2020. Rescue of SARS-CoV-2 from a single bacterial artificial chromosome. mBio 11. doi:10.1128/mBio.02168-20. PMC7520601 32978313

[17] Chiem K, Ye C, Martinez-Sobrido L. 2020. Generation of recombinant SARS-CoV-2 using a bacterial artificial chromosome. Curr Protoc Microbiol 59:e126. doi:10.1002/cpmc.126. 33048448 PMC7646048

[18] Fahnøe U, Pham LV, Fernandez-Antunez C, Costa R, Rivera-Rangel LR, Galli A, Feng S, Mikkelsen LS, Gottwein JM, Scheel TK. 2022. Versatile SARS-CoV-2 reverse-genetics systems for the study of antiviral resistance and replication. Viruses 14:172. doi:10.3390/v14020172. 35215765 PMC8878408

[19] Thi Nhu Thao T, Labroussaa F, Ebert N, V'kovski P, Stalder H, Portmann J, Kelly J, Steiner S, Holwerda M, Kratzel A, Gultom M, Schmied K, Laloli L, Hüsser L, Wider M, Pfaender S, Hirt D, Cippà V, Crespo-Pomar S, Schröder S, Muth D, Niemeyer D, Corman VM, Müller MA, Drosten C, Dijkman R, Jores J, Thiel V. 2020. Rapid reconstruction of SARS-CoV-2 using a synthetic genomics platform. Nature 582:561–565. doi:10.1038/s41586-020-2294-9. 32365353

[20] McGrath M, Xue Y, Dillen C, Oldfield L, Assad-Garcia N, Zaveri J, Singh N, Baracco L, Taylor L, Vashee S. 2022. SARS-CoV-2 variant spike and accessory gene mutations alter pathogenesis. bioRxiv. 10.1073/pnas.2204717119 PMC9477415 36040867

[21] Amarilla AA, Sng JDJ, Parry R, Deerain JM, Potter JR, Setoh YX, Rawle DJ, Le TT, Modhiran N, Wang X, Peng NYG, Torres FJ, Pyke A, Harrison JJ, Freney ME, Liang B, McMillan CLD, Cheung STM, Guevara D, Hardy JM, Bettington M, Muller DA, Coulibaly F, Moore F, Hall RA, Young PR, Mackenzie JM, Hobson-Peters J, Suhrbier A, Watterson D, Khromykh AA. 2021. A versatile reverse genetics platform for SARS-CoV-2 and other positive-strand RNA viruses. Nat Commun 12:3431. doi:10.1038/s41467-021-23779-5. 34103499 PMC8187723

[22] Torii S, Ono C, Suzuki R, Morioka Y, Anzai I, Fauzyah Y, Maeda Y, Kamitani W, Fukuhara T, Matsuura Y. 2021. Establishment of a reverse genetics system for SARS-CoV-2 using circular polymerase extension reaction. Cell Rep 35:109014. doi:10.1016/j.celrep.2021.109014. 33838744 PMC8015404

[23] Choi W-S, Jeong JH, Lloren KKS, Ahn SJ, Antigua KJC, Kim Y-i, Si Y-J, Baek YH, Choi YK, Song M-S. 2019. Development of a rapid, simple and efficient one-pot cloning method for a reverse genetics system of broad subtypes of influenza A virus. Sci Rep 9:8318. doi:10.1038/s41598-019-44813-z. 31165766 PMC6549168

[24] Quan J, Tian J. 2011. Circular polymerase extension cloning for high-throughput cloning of complex and combinatorial DNA libraries. Nat Protoc 6:242–251. doi:10.1038/nprot.2010.181. 21293463

[25] Curtis KM, Yount B, Baric RS. 2002. Heterologous gene expression from transmissible gastroenteritis virus replicon particles. J Virol 76:1422–1434. doi:10.1128/jvi.76.3.1422-1434.2002. 11773416 PMC135785

[26] Yount B, Curtis KM, Fritz EA, Hensley LE, Jahrling PB, Prentice E, Denison MR, Geisbert TW, Baric RS. 2003. Reverse genetics with a full-length infectious cDNA of severe acute respiratory syndrome coronavirus. Proc Natl Acad Sci USA 100:12995–13000. doi:10.1073/pnas.1735582100. 14569023 PMC240733

[27] Escalera A, Gonzalez-Reiche AS, Aslam S, Mena I, Laporte M, Pearl RL, Fossati A, Rathnasinghe R, Alshammary H, van de Guchte A, Farrugia K, Qin Y, Bouhaddou M, Kehrer T, Zuliani-Alvarez L, Meekins DA, Balaraman V, McDowell C, Richt JA, Bajic G, Sordillo EM, Dejosez M, Zwaka TP, Krogan NJ, Simon V, Albrecht RA, van Bakel H, García-Sastre A, Aydillo T. 2022. Mutations in SARS-CoV-2 variants of concern link to increased spike cleavage and virus transmission. Cell Host Microbe 30:373–387.e7. doi:10.1016/j.chom.2022.01.006. 35150638 PMC8776496

[28] Laha S, Chatterjee R. 2021. Temporal variations in country-specific mutational profiles of SARS-CoV-2: effect on vaccine efficacy. Future Virology 16:805–819. doi:10.2217/fvl-2021-0062. PMC8603786 34824595

[29] Panzera Y, Ramos N, Frabasile S, Calleros L, Marandino A, Tomás G, Techera C, Grecco S, Fuques E, Goñi N, Ramas V, Coppola L, Chiparelli H, Sorhouet C, Mogdasy C, Arbiza J, Delfraro A, Pérez R. 2021. A deletion in SARS-CoV-2 ORF7 identified in COVID-19 outbreak in Uruguay. Transbound Emerg Dis 68:3075–3082. doi:10.1111/tbed.14002. 33501730 PMC8014828

[30] Li Q, Wu J, Nie J, Zhang L, Hao H, Liu S, Zhao C, Zhang Q, Liu H, Nie L, Qin H, Wang M, Lu Q, Li X, Sun Q, Liu J, Zhang L, Li X, Huang W, Wang Y. 2020. The impact of mutations in SARS-CoV-2 spike on viral infectivity and antigenicity. Cell 182:1284–1294.e9. doi:10.1016/j.cell.2020.07.012. 32730807 PMC7366990

[31] Korber B, Fischer WM, Gnanakaran S, Yoon H, Theiler J, Abfalterer W, Hengartner N, Giorgi EE, Bhattacharya T, Foley B, Hastie KM, Parker MD, Partridge DG, Evans CM, Freeman TM, de Silva TI, McDanal C, Perez LG, Tang H, Moon-Walker A, Whelan SP, LaBranche CC, Saphire EO, Montefiori DC, Sheffield COVID-19 Genomics Group. 2020. Tracking changes in SARS-CoV-2 spike: evidence that D614G increases infectivity of the COVID-19 virus. Cell 182:812–827.e19. doi:10.1016/j.cell.2020.06.043. 32697968 PMC7332439

[32] Wang P, Nair MS, Liu L, Iketani S, Luo Y, Guo Y, Wang M, Yu J, Zhang B, Kwong PD, Graham BS, Mascola JR, Chang JY, Yin MT, Sobieszczyk M, Kyratsous CA, Shapiro L, Sheng Z, Huang Y, Ho DD. 2021. Antibody resistance of SARS-CoV-2 variants B.1.351 and B.1.1.7. Nature 593:130–135. doi:10.1038/s41586-021-03398-2. 33684923

[33] Gong Y-N, Tsao K-C, Hsiao M-J, Huang C-G, Huang P-N, Huang P-W, Lee K-M, Liu Y-C, Yang S-L, Kuo R-L, Chen K-F, Liu Y-C, Huang S-Y, Huang H-I, Liu M-T, Yang J-R, Chiu C-H, Yang C-T, Chen G-W, Shih S-R. 2020. SARS-CoV-2 genomic surveillance in Taiwan revealed novel ORF8-deletion mutant and clade possibly associated with infections in Middle East. Emerg Microbes Infect 9:1457–1466. doi:10.1080/22221751.2020.1782271. 32543353 PMC7473175

[34] Pacific Biosciences. 2012. Extracting DNA using phenol-chloroform. https://www.pacb.com/wp-content/uploads/2015/09/SharedProtocol-Extracting-DNA-usinig-Phenol-Chloroform.pdf.

[35] Schneider CA, Rasband WS, Eliceiri KW. 2012. NIH Image to ImageJ: 25 years of image analysis. Nat Methods 9:671–675. doi:10.1038/nmeth.2089. 22930834 PMC5554542

