# Supplementary material for: A Rapid Method for Generating Infectious SARS-CoV-2 and Variants Using Mutagenesis and Circular Polymerase Extension Cloning
Source: Microbiol Spectr. 2023 Mar 6;11(2):e03385-22. doi: 10.1128/spectrum.03385-22 (PMC10100849; doi:10.1128/spectrum.03385-22)
Supplement: Supplemental file 1 — Fig. S1 to S3 and Tables S1 and S2. Download spectrum.03385-22-s0001.pdf, PDF file, 0.4 MB [file spectrum.03385-22-s0001.pdf]

Supplementary Figure 1

A

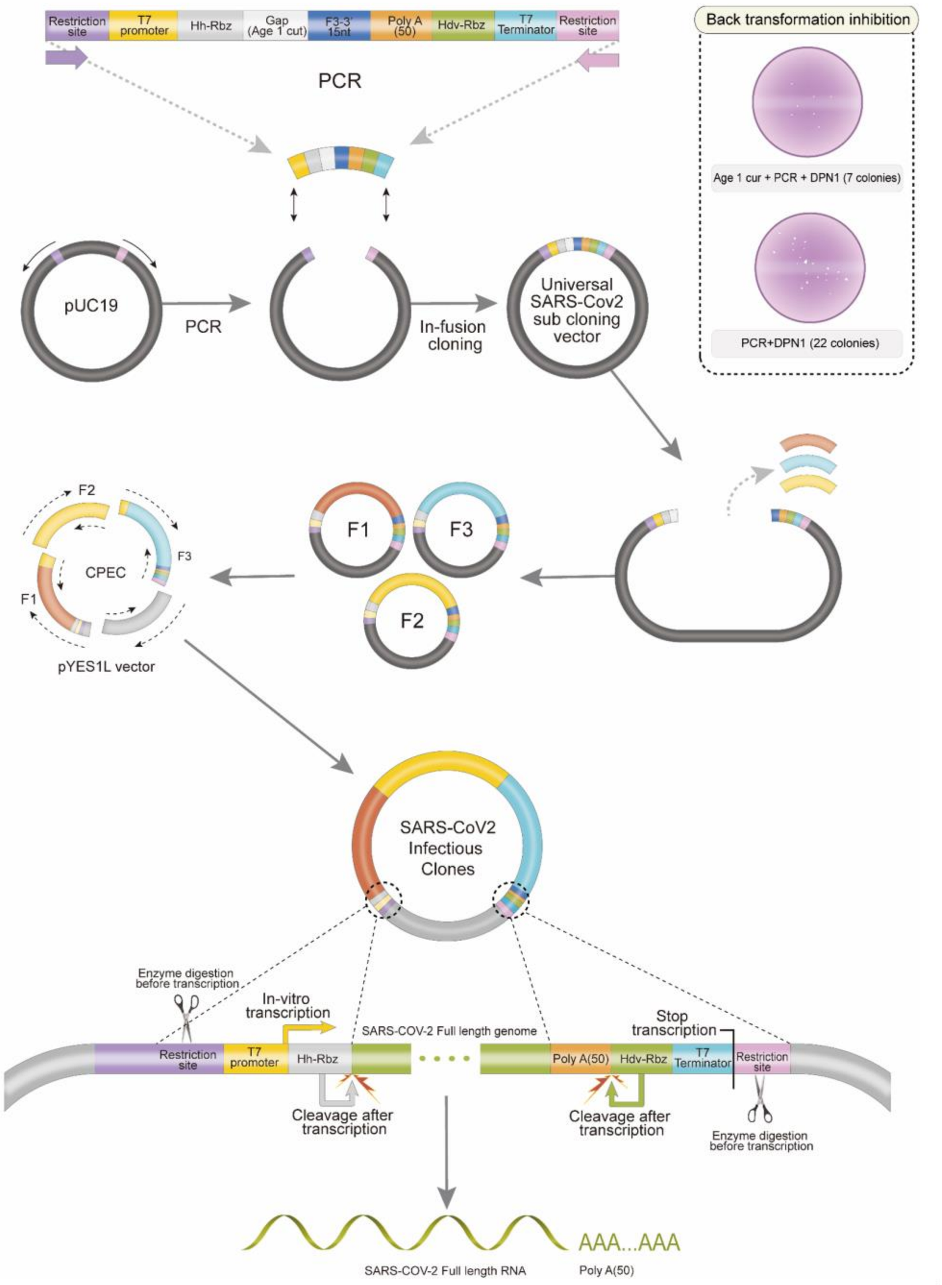

B

Nucleotide fragment	Function	Sequence(5' to3')	Base pair
Restriction site (Eag1, AsiS1, ApaI)	Added to each end of the universal insert for linearizing plasmid after assembly of whole fragments.	Front : GGGCCCTAATGCGATCGCATAC GGCCG Rear : CGGCCGTAATGGGCCCATAGCG ATCGC	27
T7 promoter	A promoter sequence which is recognized by T7 transcriptase. RNA will be synthesized by T7 promoter and T7 transcriptase.	TAATACGACTCACTATAG	18
Hh-RBZ	Hammered-Head Ribozyme is a sequence which will cut self RNA right after the sequence	GGAGAttaatCTGATGAGTCCGTG AGGACGAAACGGAGTCTAGACT CCGTC	51
Gap	Age1 resctriction enzyme site was added for enzyme digestion before making subclone, so it can decrease back transformants.	ACCGGTTCATT	11
F3-3' (15nt)	The last 15nt sequences of SARS-CoV-2 (BetaCoV/Korea/KCDC03/2020 (GISAID no. EPI_ISL_407193).	TCTTAGGAGAATGAC	15
Poly A	Poly A sequence for mimic SARS-CoV-2 genome.	AAAAAAAAAAAAAAAAAAAAAAAA AAAAAAAAAAAAAAAAAAAAAAAA AA	50
Hdv-RBZ	Hepatitis delta virus ribozyme sequences for cutting right front of the sequence of it.	GGCCGGCATGGTCCCAGCCTCC TCGCTGGCGCCGGCTGGGCAA CATTCCGAGGGGACCGTCCCCT CGGTAATGGCGAATGGGACAAC TTGTTTATTGCAGCTTATAATGGT TACAAATAAAGCAATAGCATCAC AAATTTCACAAATAAAGCATTTTT TTCACTGCATTCTAGTTGTGGTT TGTCCAAACTCATCAATGTATCTT ATCATGT	212
T7 terminator	A sequence for terminating transcription by T7 transcriptase.	CTAGCATAACCCCTTGGGGCCT CTAAACGGGTCTTGAGGGGTTT TTTG	48

Supplement Figure 1. Design and construction of SARS-CoV-2 subcloning and full-genome cloning vectors and sequence information for each functional component. A. Schematic diagram showing subcloning vector design and function in SARS-CoV-2 infectious virus recovery. Functional sequences were inserted into the pUC19 vector and subsequently used as a full-genome cloning vector for recovery of SARS-CoV-2 infectious virus. Test of efficiency of back-transformation inhibition (dash-lined square), showing fewer self–in-fusion colonies following digestion of the subcloning vector with AgeI compared with the absence of restriction digestion. B. Names of each component, sequences, and a brief description of their functions.

Supplementary Figure 2

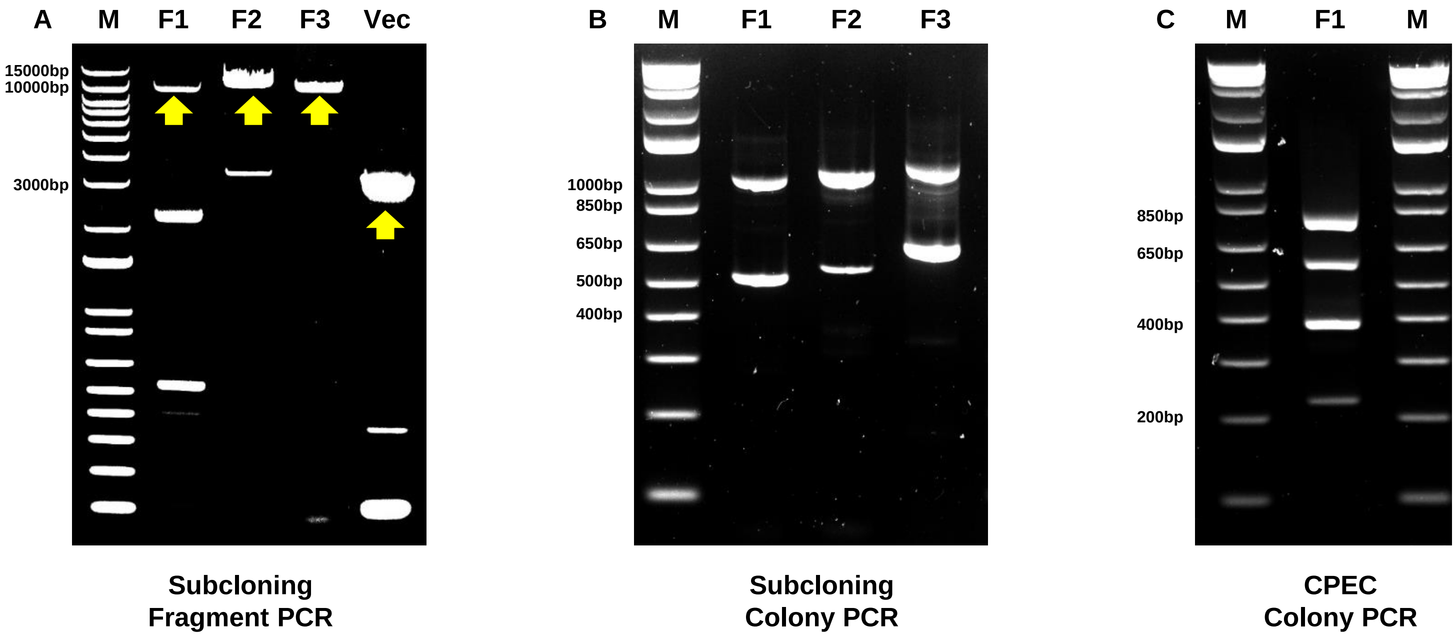

Supplement Figure 2. Gel electroporation data for SARS-CoV-2 genome PCR fragments and positive clone screening by junction PCR analysis. A. Gel electroporation of F1, F2, F3 and vector fragments for subcloning. Target fragments are indicated by yellow arrows. B Positive subclones were screened using a custom-designed subclone junction PCR method. C. Positive, full-genome clone screened using a custom-designed SARS-CoV-2 full-genome clone junction PCR method.

Supplementary Figure 3

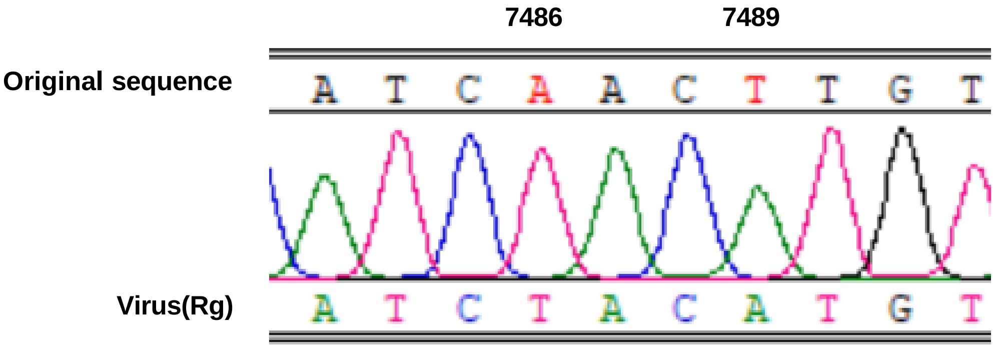

Supplement Figure 3. Discrimination of recovered recombinant SARS-CoV-2 virus from its wild-type virus. Two silent mutations, A7486T and T7489A, were introduced in recombinant SARS-CoV-2 virus as a marker for discriminating from wild-type virus.

Supplementary Table 1.

Primer name	Primer Code	Sequence (5' to 3')	Base pair	Comment
For amplification of SARS-CoV-2 subcloning fragments and vector				
Subcloning pUC-Vector ins F	SVP-1	TAATGCGATCGCATACGGCCGCGTTACATAACTTACGGTAAATGGCCCGCC	51	Primer sets for amplifying subcloning vector-insert
Subcloning pUC-Vector ins R	SVP-2	GCTATGGGCCCATTACGGCCGACATGATAAGATACATTGATGAGTTTGGA	50	
Subcloning pUC-Vector vec F	SVP-3	TAATGGGCCCATAGCGATCGCGAGCTCGAATTCACTGGCCGTCGT	45	Primer sets for amplifying subcloning vector-vector
Subcloning pUC-Vector vec R	SVP-4	GTATGCGATCGCATTAGGGCCCGCAAGCTTGGCGTAATCATGGTCATAGCT	51	
For confirmation of amplified subclones via junction PCR				
Fragment-1 Junction-1 F	FJP-1	GGATAACAATTTCACACAGGAAACAGCTATGAC	33	Primer sets to confirm amplified subclone fragment 1
Fragment-1 Junction-1 R	FJP-2	TCCAAAGCCACGTACGAGCA	20	
Fragment-1 Junction-2 F	FJP-3	ATACAGTCATGTAGTTGCCT	20	
Fragment-1 Junction-2 R	FJP-4	GTAAAACGACGGCCAGTGAATTCGAGCTCGGTA	33	
Fragment-2 Junction-1 F	FJP-5	GGATAACAATTTCACACAGGAAACAGCTATGAC	33	Primer sets to confirm amplified subclone fragment 2
Fragment-2 Junction-1 R	FJP-6	CAAAAGTACTAAAGGAAACAC	20	
Fragment-2 Junction-2 F	FJP-7	GTGAGTCTCATGGAAAACAA	33	
Fragment-2 Junction-2 R	FJP-8	GTAAAACGACGGCCAGTGAATTCGAGCTCGGTA	33	
Fragment-3 Junction-1 F	FJP-9	GGATAACAATTTCACACAGGAAACAGCTATGAC	33	Primer sets to confirm amplified subclone fragment 3
Fragment-3 Junction-1 R	FJP-10	CTACATGGCCATCTTTACACCAAAGCAT	28	
Fragment-3 Junction-2 F	FJP-11	CAACAACAAGGCCAAACTGT	20	
Fragment-3 Junction-2 R	FJP-12	GTAAAACGACGGCCAGTGAATTCGAGCTCGGTA	33	
For confirmation of amplified clones via junction PCR				
Clone Junction-1 F	CJP-1	CGTCCCTGTTTGCATTATGA	20	Primer sets to confirm amplified full-length CPEC clones via junction PCR.
Clone Junction-1 R	CJP-2	TGGTTTGTTACCTGGGAAGG	20	
Clone Junction-2 F	CJP-3	TTATAGAGTACACTGACTTT	20	
Clone Junction-2 R	CJP-4	CAAAAGTACTAAAGGAAACAC	20	
Clone Junction-3 F	CJP-5	TTGATGGACAACAGGGTGAA	20	
Clone Junction-3 R	CJP-6	TCCATTTGACTCCTGGGTTT	20	
Clone Junction-4 F	CJP-7	GCGAATGGGACAACTTGTTT	20	
Clone Junction-4 R	CJP-8	ATGTAAGCGGAGGTGTGGAG	20	

Supplementary Table 2. SARS-CoV-2 full length sequencing primers

Primer Name	Sequence (5' to 3')	Primer binding site	Base pair
1F	ATTAAAGGTTTATACCTTCCCAGG	1–24	24
800R	TCTTGAAAATCTTCATAAGGATCAGTG	715–741	27
1300F	AACTTCATGGCAGACGGGCG	1249–1268	20
1500R	GTCACCAACAATATTGATGTTGA	1641–1663	23
1400F	AACCATACAGGTGTTGTTGG	1565–1584	20
2600F	GGAAGTTGTCTTGAAAACTG	2527–2546	20
2900R	TCACCTTCTTCTTCATCCTC	3050–3069	20
2800F	TTAGATGAGTGGAGTATGGC	2954–2973	20
4000F	TGAAAGTAAACCTTCAGTTG	3913–3932	20
4300R	CGCTGTATAGTTGAAACTATGGC	4454–4476	23
4200F	GGAACTGTTTCTTGGAATTTGCGAG	4370–4394	25
5300F	AGGTACATGTCAGCATTAAA	5198–5217	20
5700R	GAAAACATCCGTAATAGGAC	5847–5866	20
5600F	CCAGTGTGGTCACTATAAAC	5758–5777	20
6600F	CACACAGATCTAATGGCTGC	6539–6558	20
7100R	CCAAGTACATAGAAAAACCTAGTG	7267–7290	24
7000F	CTTTAGACACCTATCCTTC	7155–7173	19
8300F	TTGTTGATTCAGATGTAGAA	8187–8206	20
8500R	CCTATGATTTCACTTGAAAAG	8668–8688	21
8400F	ACTTAAGGGTGGTAAAATTGTTAAT	8542–8566	25
9600F	ATACAGTCATGTAGTTGCCT	9484–9503	20
9900R	TTTACCAGATGGGAATGCC	10072–10090	19
11300F	TCTCTTGCCACTGTAGCTTA	11192–11211	20
11500R	CAAGAGTCAGTCTAAAGTAGCGG	11668–11690	23
12200R	TGTTGTCCAGCATTTCTTCA	12058–12077	20
11400F	GCCCTATTTTCTTCATAACTGG	11562–11583	22
12900R	GCATCTACAGCAAAAGCACAG	13072–13092	21
12800F	GGATTAAACAACCTAAATAGAGG	12962–12984	23
14000F	GGCAGACCTCGTCTATGCTT	13812–13831	20
14300R	CTCTCTGAAGTGGTATCCAGTTG	14468–14490	23
14200F	CAGATGCATTCTGCATTGTG	14352–14371	20
15700R	GAGAGCAAAATTCATGAGGTCC	15862–15883	22
15600F	CTCAAGGTCTAGTGGCTAGCA	15755–15775	21
17100R	TCAAAACACTCTACACGAGC	17248–17267	20
17000F	AGCTTGCTCTCATGCCGCTGTT	17157–17178	22
18500R	TGGCACGTCTATCACATAGA	18663–18682	20
18400F	CAAAATCTCTCTGACAGAG	18561–18580	19
19800F	ATGTGGCTTTTAATGTTGTA	19631–19650	20
19900R	GGACCTACAGATGGTTGTA	20072–20090	19
21200F	GTGATATGTACGACCCTAAG	21044–21063	20
21500R	CAGGGTAATAAACACCACGTGTG	21658–21680	23
21400F	TTGTTTTTCTTGTTTTATTGCC	21567–21588	22
22700F	AGAGTCCAACCAACAGAATC	22517–22536	20
22900R	CCAACACCATTAGTGGGTTGG	23053–23073	21
22800F	GAAATCTATCAGGCCGGTAGC	22973–22993	21
24300R	CACTTGAAATTGCACCAAAATTGGAGC	24462–24488	27
24200F	GTGCTATTGGCAAAATTCAAG	24348–24368	21
25700R	CACTATTGTAAGGTATACAATAGTCG	25854–25879	26
25600F	ATGAGGCTTTGGCTTTGCTGG	25765–25785	21
27100R	CCAAATGGAAACTTTAAAAGTCCTC	27258–27282	25
27000F	CCATTCCAGTAGCAGTGACAATATTG	27149–27174	26
28300F	CACCCATTCAGTACATCGAT	28099–28118	20
28500R	AAGGCTCCCTCAGTTGCAAC	28670–28689	20
28400F	CGTGGTGGTGACGGTAAAATG	28556–28576	21
29200F	CAACAACAAGGCCAAACTGT	28991–29010	20
29851R	GTCATTCTCCTAAGAAGCTA	29851–29870	20
